# Mammary Fibrosis Tendency and Mitochondrial Adaptability in Dairy Cows with Mastitis

**DOI:** 10.3390/metabo12111035

**Published:** 2022-10-28

**Authors:** Xingchi Kan, Guiqiu Hu, Yiyao Liu, Ping Xu, Yaping Huang, Xiangyu Cai, Wenjin Guo, Shoupeng Fu, Juxiong Liu

**Affiliations:** 1 Key Laboratory of Zoonoses Research, Ministry of Education, College of Veterinary Medicine, Jilin University, 5333 Xi’an Road, Changchun 130062, China; 2 Zhijiang Laboratory, Kechuang Avenue, Hangzhou 311121, China

**Keywords:** mastitis, mammary fibrosis, TGF-β1, mitochondrial damage, ROS

## Abstract

Dairy cow mammary gland fibrosis causes huge economic losses to livestock production, however, research on dairy cow mammary gland fibrosis is in its infancy and it lacks effective treatments. Therefore, the purpose of this experiment was to explore the correlation between mastitis and fibrosis and mitochondrial damage, and to further explore its pathogenesis. In vivo, mammary tissue and milk samples were collected from healthy cows (*n* = 10) and mastitis cows (*n* = 10). The results of the study showed that compared with the control group, the mastitis tissue showed tissue damage, accumulation of collagen fibers, and the content of TGF-β1 in mammary tissue and milk was significantly increased; the level of inflammatory mediators was significantly increased; the fibrotic phenotype, collagen 1, α-SMA, vimentin gene, and protein levels were significantly increased, while the E-cadherin gene and protein levels were significantly decreased. In vitro, based on TGF-β1-induced bMECs, the above experimental results were further confirmed, and TGF-β1 significantly promoted the fibrotic phenotype of bMECs. On the other hand, in vivo results showed that fibrotic mammary tissue had a significantly stronger mitochondrial damage phenotype and significantly higher ROS than the control group. In vitro, the results also found that TGF-β1 induced a significant increase in the mitochondrial damage phenotype of bMECs, accompanied by a large amount of ROS production. Furthermore, in a TGF-β1-induced bMEC model, inhibiting the accumulation of ROS effectively alleviated the elevated fibrotic phenotype of TGF-β1-induced bMECs. In conclusion, the fibrotic phenotype of mammary gland tissue in dairy cows with mastitis was significantly increased, and mastitis disease was positively correlated with mammary fibrotic lesions. In an in vitro and in vivo model of cow mammary fibrosis, bMECs have impaired mitochondrial structure and dysfunction. Inhibiting the accumulation of ROS effectively alleviates the elevated fibrotic phenotype, which may be a potential therapeutic approach to alleviate mammary fibrosis.

## 1. Introduction

Mammary fibrosis in dairy cows seriously shortens the lactation period and reduces milk yield and milk quality. This pathological process causes huge economic losses to animal husbandry [1,2]. In clinical production practice, cow mammary gland fibrosis is a serious pathological process. However, there is a lack of specific experimental data on the harm of mammary fibrosis in dairy cows, therefore, it is very urgent to study the pathogenesis of cow mammary fibrosis.

The epithelial–mesenchymal transition (EMT) process produces a large amount of extracellular matrix (ECM), and ECM is the main cause of fibrosis [1,3]. Therefore, EMT has been recognized as an important factor of fibrotic disease [4,5,6]. During EMT, epithelial cells morphologically change and acquire certain features of surrounding stromal cells [1], such as the loss of epithelial cell polarity, epithelial cells from cobblestone-like to long-spindle-like showing a fibroblast-like appearance, and typical phenotypic changes are increased levels of α-SMA, vimentin, fibronectin, and decreased E-cadherin protein level [7]. Epithelial cells exhibit functional similarity to fibroblasts after EMT [1]. However, there are relatively few studies on mammary fibrosis in dairy cows, and further research is still needed.

A cell contains about 1000–2000 mitochondria [8]. Mitochondria are the place where cells carry out aerobic respiration, and they are also the energy factories of cells. The integrity of mitochondrial structure and normal function is an important factor in maintaining cell homeostasis [9,10]. However, when mitochondria were under high metabolic pressure, they were easily damaged by stimulation [9]. Studies have shown that mammary epithelial cells exhibit mitochondrial damage and dysfunction during mastitis [11,12]. Mitochondrial damage is often closely related to fibrotic processes, such as liver fibrosis [8], kidney fibrosis [13], pulmonary fibrosis [14,15], etc. Studies have shown that the release of a large amount of ROS from mitochondrial damage is often an important factor in inducing the development of fibrosis. In addition, studies have shown that inhibiting the accumulation of ROS is an effective treatment for alleviating lung and liver fibrosis [16,17]. Therefore, effectively alleviating mitochondrial damage and inhibiting the accumulation of ROS may be potential therapeutic approaches to alleviate the fibrotic process. So, whether mitochondria are damaged in the process of mammary fibrosis and whether alleviating mitochondrial damage is expected to be a potential treatment for mammary fibrosis have not yet been reported. Therefore, the purpose of this experiment was to explore the correlation between mastitis and mammary fibrosis and mitochondrial damage, in order to provide experimental data and theoretical guidance for the prevention and treatment of mammary fibrosis in dairy cows.

## 2. Materials and Methods

### 2.1. Animals

This experiment was approved and supported by the Animal Welfare Ethics Committee of Jilin University (SYXK(Ji)2016-0001). For the scientificity and rigor of the experiment, the animal experiment process abided by the following standards. All cows ingested the same diet and Table 1 shows the detailed composition of the cows’ diet. Dairy cows had free access to feed and water. The cows used in the experiment were all from Guangze Ecological Ranch in Jilin Province, China, which is the production–university–research cooperation base and teaching experiment base of the College of Veterinary Medicine of Jilin University. This experiment randomly selected cows with similar years of lactation (median = 3, range = 2–4), DIM (median = 6 d, range = 3–9 d), and parity. The control group was cow mammary gland tissue with good physiological function, and the number of somatic cells was controlled within the range of 20,000–200,000/mL. In the control group, there was no human infection or natural infection of pathogenic microorganisms in cow mammary gland tissue. In the model group, the infection of cow mastitis was the same. The type of disease in the model group was a naturally infected E. coli-type dairy mastitis. The clinical cow mastitis tissue was used as the experimental object, and the clinical cow mastitis was divided into 3 grades. The specific grading standards were as follows (Table 2. Grade 1: There was a small number of milk lumps in the milk, and there was no obvious redness, swelling, heat, and pain in the mammary area; Grade 2: There were a lot of milk lumps in the milk, obvious separation of water and milk, and slight redness, swelling, heat, and pain in the mammary area, etc.; Grade 3: The cows showed symptoms of general discomfort, increased body temperature, watery milk, and severe redness, swelling, heat, and pain in the milk area. The mammary gland tissue and milk of Grade 2 mastitis were selected in this experiment, and the cows used in the experiment were selected by three experienced professional veterinarians. The information of cows involved in the experiment was shown in Table 3 and Table 4.

### 2.2. Collection of Samples

50 mL of fresh milk was collected with a centrifuge tube. When the cow finished in the milking workshop, the milking worker held the milk sample in 50 mL centrifugal tubes. The number on the dairy cows’ ears was the main method for identifying dairy information. Before collection of mammary tissue samples, 2% lidocaine hydrochloride was injected subcutaneously around the sampling site. Iodine and 75% alcohol were used for adequate disinfection of the skin surface. The prepared sterile veterinary puncture needle was then inserted into mammary gland tissue, and an appropriate amount of mammary gland tissue was collected and stored in liquid nitrogen or 4% formaldehyde solution. During the experiment, the cows were placed in a convenient and fixed enclosure.

### 2.3. Determination of TGF-β1 in Milk

Fifty milliliters of milk was centrifuged at 3000 rpm for 20 min. The milk supernatant was collected to avoid inhalation of milk fat during collection. The experimental steps were strictly in accordance with the instructions of the enzyme-linked kit (Enzyme-Linked Biotechnology Co., Ltd., Shanghai, China). Forty microliters of whey samples and standards was added to a 96-well plate, and a control well was set. The 96-well plate was coated with TGF-β1 antibody in advance. One hundred microliters of horseradish peroxidase was added to each well and incubated at 37 °C for 60 min. After washing, 100 μL of chromogenic liquid was added and incubated at 37 °C for 15 min. 50 μL of dilute sulfuric acid solution was added to each well, and finally the experimental results were measured at 450 nm.

### 2.4. H&E Staining

Fresh mammary tissue was immersed in 4% formaldehyde solution for 48 h. Immediately after the mammary gland tissue was dehydrated in a gradient of alcohol solution, soaked in xylene solution, and embedded in paraffin, slices with a thickness of 5 μm were obtained. The sections were dried for H&E staining, and the specific steps were as follows. Sections were dewaxed with xylene, dehydrated with graded alcohol, passed through hematoxylin and eosin staining solution (Solebo, Beijing, China) in turn, and finally mounted with neutral resin dropwise.

### 2.5. Masson Staining

A Masson Kit (G1340, Solarbio Science & Technology Co., Ltd., Beijing, China) was used. (1) Dimethylbenzene was used for dewaxing of pathological slices of mammary tissue. (2) Weigert iron sumulin staining solution was used for treatment of mammary slices for 5 min. (3) Acidal ethanol differentiation liquid was used for treatment of slices for 10 s. (4) Masson Blue Liquid was used for treatment of slices for 4 min, and then washed with distilled water for 1 min. (5) Lichun red staining solution was used for treatment of slices for 7 min. (6) Slices were washed with weak acid working liquids for 1 min, (weak acid working liquid = 2 times volume of distilled water + 1 times the volume of weak acid solution). (7) Phosphomolybdic acid solution was used for treatment of mammary slices for 2 min. (8) Aniline blue dyeing solution was used for treatment of slices for 2 min. (9) Water-free ethanol was used for treatment of mammary slices 3 times, 5 s each time. (10) Dimethylbenzene was used for treatment of mammary slices for 3 min, and closed with neutral gum. Finally, the experimental results were measured under an optical microscope and the picture results were obtained.

### 2.6. Isolation and Culture of bMECs

As described in previous work, the isolation of primary cow mammary epithelial cells (bMECs) was performed in tissue block culture [18]. Mammary gland tissue from healthy cows at peak lactation was used for the isolation of bMECs. The mammary gland tissue was derived from fresh tissue from a dairy cow slaughterhouse, and the tissue with good mammary gland physiological function was selected. Cells were isolated in the fall and winter to increase the success rate of the experiment. The fresh mammary tissue block was rinsed three times with 75% alcohol and PBS in order to remove the milk inside the mammary tissue. The mammary tissue was transferred to an ultra-clean bench, and a small piece of deep mammary tissue of about 2 mm was removed with sterile scissors, avoiding the larger milk ducts. Small pieces of mammary gland tissue were placed in sterile glass bottles with a volume of 4 mL and submerged in DMEM containing 10% FBS, and the mammary gland tissues were minced to 1 mm size with small scissors. The chopped mammary gland tissue was transferred into a 25 cm cell culture flask, and the culture flask was placed upside down for 3 h to eliminate the moisture in the mammary gland tissue, which was conducive to firmly sticking to the bottom wall of the bottle, and then 5 mL was added to the culture flask. After 3 days, fibroblasts first grew around the mammary tissue, and 0.25% trypsin was used to digest and purify bMECs. After 5 days, epithelial cells began to grow around the mammary tissue block, and bMECs were collected for subsequent experiments. bMECs were placed in a cell culture incubator at 37 °C with 5% CO_2_ [19,20]. The time for TGF-β1 to stimulate bMECs in the experiment was 48 h.

### 2.7. Determination of Myeloperoxidase (MPO)

Fresh mammary tissue was put into a 2 mL grinding tube, and 2 mL of HEPS solution was added per 0.5 g of tissue. Seven sterile steel balls with a diameter of 2 mm were placed in the centrifuge tube, and it was ground in a tissue grinding apparatus at a frequency of 220 V 50 Hz for 10 min. Immediately after centrifugation at 12,000 rpm for 10 min, an equal volume of 0.5% CTAC solution was added to the pellet to continue grinding for 10 min. Finally, the supernatant solution was the MPO detection sample, which was diluted 10 times with CTAC solution during MPO detection.

The specific MPO experimental procedure was as described previously [21]. First, 8798 μL of 3 mM TMB, 180 μL of 6 mM resorcinol, and 22.5 μL of 3% hydrogen peroxide were used as detection substrates to detect 80 samples. Then, 75 μL of MPO in each well was reacted with 75 μL of substrate for 2 min, 2 mM H_2_SO_4_ was added to stop the reaction, and the absorbance at 450 nm was detected with a microplate reader. The number of experimental samples was the mammary gland tissue of 10 cows in each group, and 3 technical replicates were performed for each cow.

### 2.8. qRT PCR Assay

Extraction of total RNA from bMECs [22] involved 1 mL Trizol (Sigma-Aldrich, Saint Louis, MO, USA) added to tissues or cells, then 200 μL of chloroform was added and shaken vigorously for 30 s, 500 μL of isopropanol was added and left to stand for 30 min, 1000 μL of 75% DEPC alcohol was added, and finally 20 μL of DEPC water was added. The concentration of total RNA was measured using a NanoDrop 2000C instrument, the total RNA quality score of 260/280 of all samples was controlled within the range of 1.9~2.0 (Thermo Scientific, Beijing, China). Experimental procedures for reverse transcription were carried out according to the manufacturer’s instructions. Specific cycle temperatures were 70 °C for 10 min, 42 °C for 60 min, and 70 °C for 15 min.

qRT-PCR experimental operation involved the primer CDS sequences being downloaded from the NCBI GenBank database, and primers were designed on the Shanghai Sangon Biology website. cDNA was amplified in a 20 μL system. The specific components were 10 μL SYBR green, 1 μL each of upstream and downstream primers, and 8 μL 10-fold diluted cDNA template. β-actin was used as an internal reference in this experiment because its expression is stable in normal and inflamed mammary glands [23,24]. Each group contained mammary gland tissue from 10 cows, and 3 technical replicates were performed per cow. The detailed primer information is shown in Table 5.

### 2.9. Western Blotting

For determination of the protein concentration of bMECs and mammary gland tissues, the BCA protein kit (Shanghai Biyuntian, Shanghai, China) was used. Protein was sampled with a standard of 35 mg/15 μL. Separation was performed on 12% SDS-PAGE gel at 75 V for 30 min and 100 V for 60 min. Proteins on the SDS-PAGE gel were transferred to a PVDF membrane by wet transfer technique at 75 V for 80 min. PVDF membranes were blocked with 20% milk for 2 h. The primary antibody was diluted in 20% BSA and kept at 4 °C overnight. On the second day, the secondary antibody was diluted with 20% milk according to a certain ratio, and the PVDF membrane was incubated for 1 h. Finally, the supersensitive luminescent solution was added dropwise on the PVDF membrane, and the exposure information of the protein band was obtained by X-ray film in a darkroom.

The primary antibody information is as follows: iNOS (1:1000, abcam, ab15323, Tokyo, Japan), collagen 1 (1:1000, abconal, a16891, Wuhan, China), E-cadherin (1:2000, Affinity, af0131, Shanghai, China), α-SMA (1:3000, Affinity, AF1032) Vimentin (1:1000, Affinity, af7013), Mfn2 (1:2000, proteintech, Wuhan, China), cytochrome C (1:500, proteintech, 10993-1-ap, Wuhan, China), VDAC1 (proteintech, 55259-1-ap), HSP60 (proteintech, 15282-1-ap), collagen 2 (1:2000, Santa, SC-52658, Santa Cruz Biotechnology, Santa Cruz, CA, USA), TFAM (1:200, Santa, sc166965), β-actin (1:5000, Solarbio, K200058M). The secondary antibody information is as follows: Goat antimouse IgG (1:5000, ba1051, Boster Bioengineering Co., Ltd., Wuhan, China) and sheep antirabbit IgG (1:5000, ba1055).

### 2.10. Immunofluorescence Measurement

The immunofluorescence method was used for the detection of bMEC-specific marker CK-18. (1) When the cell density reached about 70%, the cells were taken out of the incubator and rinsed with PBS 3 times, 5 min each time. (2) Fixation: Cells were fixed in 4% formaldehyde solution for 60 min. (3) Cell permeabilization: 0.1% Triton × −100 treatment for 15 min. (4) Immunofluorescence blocking: Blocked with 5% donkey serum for 2 h, cells were rinsed with PBS 3 times, 5 min each time. (5) Incubation of the first antibody: The primary antibody (Cytokeratin 18, 1:200, proteintech, Wuhan, China) solution was immersed overnight at 4 °C. (6) Incubation of the second antibody: 1 μL of secondary antibody solution (donkey antirabbit IgG (H + L) Highly Cross-Adsorbed Secondary Antibody, Alexa Fluor 488) was added to 5% donkey serum, and the secondary antibody solution was immersed for 1 h at room temperature. (7) DAPI was added dropwise to the cell surface for 2 min. (8) The coverslip was closed with glycerin. The experimental results were observed under a fluorescence microscope.

### 2.11. MitoTracker Staining

A MitoTracker Red CMXRos kit was purchased from Beyotime Biotechnology Co., Ltd. (C1049B, Wuhan, China) in order to carry out MitoTracker staining. After stimulating bMECs with 5 ng/mL TGF-β1 for 48 h. (1) Preparation of storage: 50 μL Mito-Tracker Red CMXROS solution was added to 420 μL of water-free anhydrous dimethyl sulfoxide (DMSO). After mixing, the storage solution with a concentration of 200 μm was obtained, and stored at −20 °C. (2) Preparation of the working solution: A small amount of 200 μm storage solution was added to the cell culture solution at a ratio of 1:1000, so that the final concentration was 20 nm, and the working liquid was heated to 37 °C before use. (3) When the cells were cultivated to a certain density in the cell culture plate or a Petri dish, the cell culture medium was removed, the prepared work solution was added, and incubated for 15 min at 37 °C. (4) The working liquid was removed and fresh cell culture liquid was added with pretemperature cultivation at 37 °C. (5) The results were observed or detected with a fluorescent microscope.

### 2.12. Mitochondrial Membrane Potential JC-1 Detection

In order to assess mitochondrial membrane potential, a JC-1 kit was purchased from Beyotime Biotechnology Co., Ltd. (C2005, Wuhan, China). (1) Preparation of JC-1 dyeing work solution: JC-1 (200×) was diluted by ultra-pure water to 1 × JC-1 staining working solution. (2) For a hole in a six-hole board, the culture medium was removed, and 1 mL JC-1 staining working solution was added and kept at 37 °C in the cell culture box for 20 min. (3) During the incubation period, an appropriate amount of JC-1 staining buffer (1×) was made and placed in an ice bath. (4) After the incubation at 37 °C, the sample was removed and washed with JC-1 staining buffer (1×) twice. (5) Two milliliters of cell culture liquid was added, which can contain serum and phenol red in the culture solution. (6) Fresh microscopy was carried out. When detecting the JC-1 signal, the excitation was set to 490 nm, and the transmitting light was set to 530 nm.

### 2.13. Detection of ROS Content in Mammary Gland

In order to further explore the oxidation level of mammary tissue, a Reactive Oxygen Species Assay Kit was purchased from Beyotime Biotechnology Co., Ltd. (S0033S, Wuhan, China). Briefly, 1 g of mammary gland tissue was added to 10 mL of collagenase (2 mg/mL, Solarbio Science & Technology Co., Ltd., C8140, Beijing, China). The collagenase contained 1/1000 of DCFH-DA. The mammary gland tissue was minced and placed at 37 °C for 60 min. The tissue suspension was treated with a 40 μM filter, and an equal amount of the suspension was drawn. The cell suspension was centrifuged at 3000 rpm for 3 min, and the supernatant cell pellet was removed and washed three times with DMEM. After the cell suspension was diluted 10 times, the fluorescence value of ROS was measured with a microplate reader (excitation wavelength: 488 nm; emission wavelength: 525 nm). After the cells were stimulated, 100 μL of DCFH-DA was added to each well to make the final concentration 10 μM. Cells were incubated at 37 °C for 20 min, rinsed with PBS, and the fluorescence value of ROS was measured with a microplate reader.

### 2.14. Detection of ROS Content in bMECs

The ROS scavenging reagent NAC (3 mM, Solarbio Science & Technology Co., Ltd., Beijing, China) was used to pretreat bMECs for 1 h. Then, the DCFH-DA probe was diluted in DMEM to a final concentration of 10 μM/L. The DCFH-DA probe solution was immersed in the treated bMECs and the cells were kept at 37 °C for 30 min. Cells were washed three times with serum-free cell culture medium to sufficiently remove DCFH-DA that did not enter the cells. Cells were digested with 0.25% trypsin and centrifuged at 2500 rpm to reconstitute the cell suspension in PBS. Flow cytometry was used to detect the level of ROS produced by bMECs.

### 2.15. Data and Statistical Analysis

**Figure 1:**
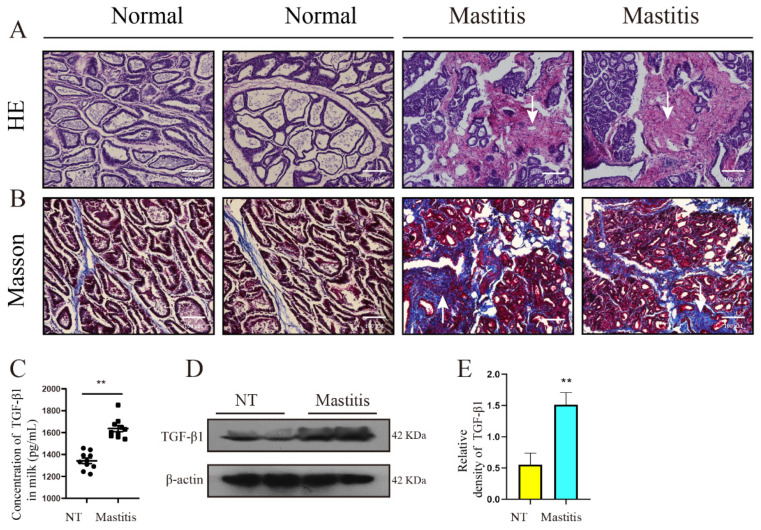
Mammary histology and TGF-β1 content in milk. (**A**,**B**) H&E staining and Masson results of mammary tissues of healthy cows (*n* = 10) and cows with mastitis, respectively (*n* = 10). (**C**) The concentration of TGF-β1 in the milk of healthy dairy cows (*n* = 10) and mastitis dairy cows (*n* = 10). (**D**) The protein levels of TGF-β1 and β-actin in the mammary gland tissues of healthy dairy cows (*n* = 10) and mastitis dairy cows (*n* = 10). (**E**) Quantification of TGF-β1 protein levels in mammary tissue, relative to β-actin. Scale bar 100 μm. (**C**) is the result of the Wilcoxon signed rank test method. The data are represented by the median and interquartile range (IQR). Each point represents an individual sample, the center line is the median, and the boundary line represents the 25th percentile and the interquartile range, respectively. (**E**) is the result of the unpaired *t*-test method. The data error is presented in the form of mean ± SEM. The results of the experiment are the result of three independent repeated experiments. ** *p* < 0.01 compared with the control group.

GraphPad Prism 8 or SPSS software (IBM, Chicago, IL, USA) was used for analysis of experimental data. Figure 1C is the result of the Wilcoxon signed rank test method. The data are represented by the median and interquartile range (IQR). Each point represents an individual sample, the center line is the median, and the boundary line represents the 25th percentile and the interquartile range, respectively. qRT-PCR, Western blot, and other experimental data were analyzed using the unpaired *t*-test statistical method. Adobe Illustrator (JLU, Changchun, Jilin, https://zbhrj1.jlu.edu.cn/download/Adobe_Illustrator.html, accessed on 1 September 2021.) software was used for the combination of experimental pictures. The data error is presented in the form of mean ± SEM. The results of the experiment were the result of three independent repeated experiments.

## 3. Results

### 3.1. Mammary Histology and Changes of TGF-β1 Content

To evaluate the effect of cow mastitis on mammary gland fibrosis, we obtained the following experimental results. H&E results showed that mastitis resulted in severe destruction of mammary tissue and massive collagen deposition in the interstitial spaces, as indicated by the arrows in the pink area (Figure 1A). Masson results also showed that mastitis resulted in the deposition of a large number of collagen fibers in mammary tissue, with dark blue representing collagen fibers (the main cause of fibrosis) and dark red representing muscle fibers (Figure 1B). TGF-β1 is the main stimulatory factor that promotes the occurrence and development of the pathological process of fibrosis, and its level reflects the strength of the fibrosis process. The ELISA results showed that the content of TGF-β1 in the mammary milk of mastitis was significantly higher than that of the control group (Figure 1C, *p* < 0.01). WB results showed that the content of TGF-β1 in mastitis tissue was significantly higher than that in control group (Figure 1D,E, *p* < 0.01). These results showed that TGF-β1 was massively secreted and collagen was deposited in mastitis tissue, which preliminarily confirmed the occurrence of mammary tissue fibrosis.

### 3.2. Levels of Proinflammatory Mediators in Mammary Tissue of Healthy Dairy Cows and Mastitis Dairy Cows

**Figure 2:**
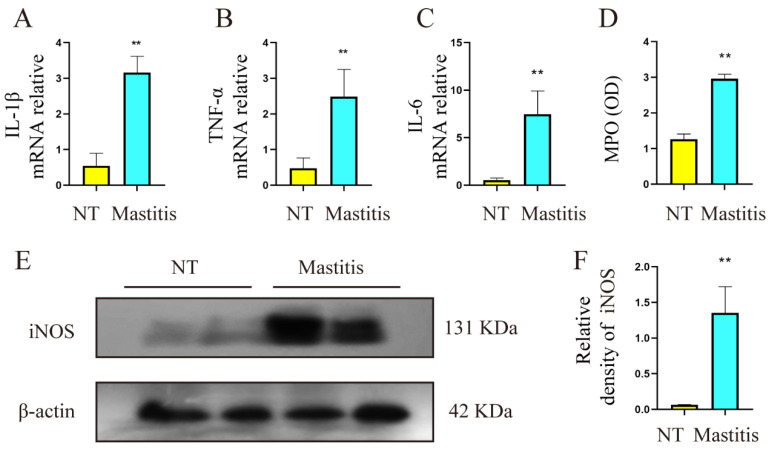
Levels of proinflammatory mediators in mammary tissue of healthy dairy cows (*n* = 10) and mastitis cows (*n* = 10). (**A**–**C**) The mRNA expression levels of IL-1β, TNF-α, IL-6 in mammary tissues. (**D**) The relative change trend of MPO in mammary tissues. (**E**) The protein levels of iNOS and β-actin in the mammary gland. (**F**) Quantification of TGF-β1 protein levels in mammary tissue, relative to β-actin. Data were analyzed by the unpaired *t*-test method. The data error is presented in the form of mean ± SEM. The results of the experiment are the result of three independent repeated experiments. ** *p* < 0.01 compared with the control group.

The following experimental results were obtained to assess inflammation in healthy and mastitis mammary gland tissue of cows. The mRNA results showed that the contents of inflammatory mediators IL-1β, IL-6, and TNF-α in mastitis tissues were significantly higher than those in the control group (Figure 2A–C, *p* < 0.01). The MPO results showed that the level of myeloperoxidase (MPO) in the mastitis tissue was significantly higher than that in the control group (Figure 2D, *p* < 0.01). The protein results showed that the level of inducible nitric oxide synthase (iNOS) in the mastitis tissue was significantly higher than that in the control group (Figure 2E,F, *p* < 0.01). These results suggest that there is a higher inflammatory state in mastitis tissue, providing a favorable environment for the development of mammary fibrosis.

### 3.3. Changes of Fibrotic Phenotype in Mammary Glands of Mastitis Cows

**Figure 3:**
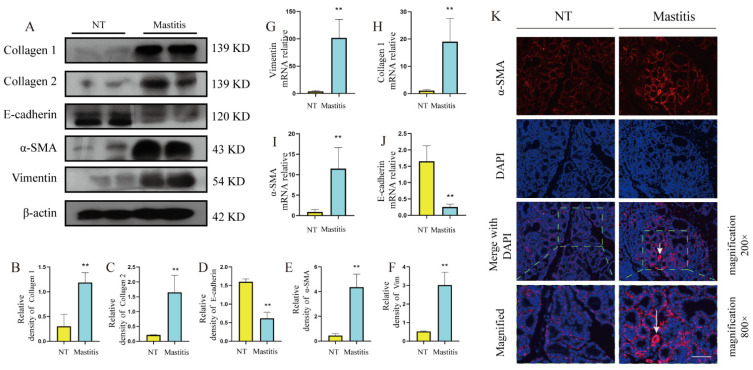
Phenotypic changes of fibrosis in mammary gland tissue of healthy dairy cows (*n* = 10) and mastitis dairy cows (*n* = 10). (**A**) The protein levels of collagen 1, collagen 2, E-cadherin, α-SMA, vimentin, and β-actin in the mammary gland. (**B**–**F**) Quantification of collagen 1, collagen 2, E-cadherin, α-SMA, vimentin protein levels in mammary tissue, relative to β-actin. (**G**–**J**) The mRNA expression levels of vimentin, collagen 1, α-SMA, E-cadherin, in mammary tissues. (**K**) Immunofluorescence results of α-SMA, scale bar 200 μM. Data were analyzed by the unpaired *t*-test method. The data error is presented in the form of mean ± SEM. The results of the experiment are the result of three independent repeated experiments. ** *p* < 0.01 compared with the control group.

To assess changes in fibrosis-related phenotypes in mastitis tissue, we obtained the following experimental results. The results of protein study showed that the levels of collagen 1 and collagen 2, the main components of the ECM, were significantly higher than those of the control group (Figure 3A–C, *p* < 0.01). The gene results also showed that the mRNA level of collagen 1 was significantly higher than that of the control group (Figure 3H, *p* < 0.01). The protein study results showed that the levels of fibroblast activation markers α-SMA and vimentin were significantly higher than those of the control group (Figure 3A,E,F, *p* < 0.01), and the gene results also showed that the mRNA levels of α-SMA and vimentin were significantly higher than those of the control group (Figure 3G,I, *p* < 0.01). E-cadherin is a calcium-dependent transmembrane protein involved in cell adhesion. It is distributed in various epithelial cells and plays an important role in maintaining cell polarity and integrity. The protein study results showed that the level of E-cadherin was significantly lower than that of the control group (Figure 3A,D, *p* < 0.01), and the gene results also showed that its mRNA level was significantly lower than that of the control group (Figure 3J, *p* < 0.01). These results indicate that a large amount of ECM accumulates in mastitis tissue, the fibrosis process was activated, and the tight junction structure between cells was destroyed, which ultimately drives the occurrence of fibrotic lesions in mammary tissue.

### 3.4. Mitochondrial Damage in Fibrotic Mammary Tissue

**Figure 4:**
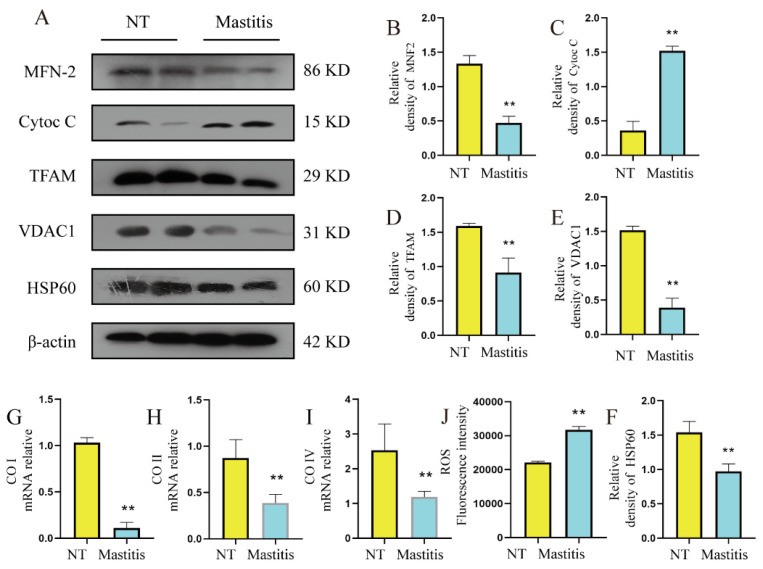
Mitochondrial damage in fibrotic mammary tissue. (**A**) The protein levels of MFN2, Cyto C, TFAM, VDAC1, HSP60, and β-actin in the mammary gland. (**B**–**F**) Quantification of MFN2, Cyto C, TFAM, VDAC1, HSP60 protein levels in mammary tissue, relative to β-actin. (**G**–**I**) The mRNA expression levels of COI, COII, COIV, in mammary tissues. (**J**) ROS content in mammary gland tissue of dairy cows. Data were analyzed by the unpaired *t*-test method. The data error is presented in the form of mean ± SEM. The results of the experiment are the result of three independent repeated experiments. ** *p* < 0.01 compared with the control group.

The lactation capacity of the mammary gland is inseparable from the energy supply of mitochondria. In order to explore the relationship between mammary fibrosis lesions and mitochondria, we detected changes in mitochondrial damage in fibrotic mammary tissue, and obtained the following experimental results. The protein results showed that the protein levels of HSP60 (major marker in mitochondria) and voltage-dependent anion selective channel (VDAC1, marker of mitochondrial outer membrane) in mammary tissue were significantly lower than those in the control group (Figure 4A,E,F, *p* < 0.01), suggesting that mitochondrial numbers were reduced in fibrotic mammary tissue. The protein level of MFN2 in mammary tissue was significantly lower than that in the control group (Figure 4A,B, *p* < 0.01), which suggested that mitochondrial fusion capacity was weakened in fibrotic mammary tissue. The protein level of Cytoc C was significantly higher than that in the control group (Figure 4A,C, *p* < 0.01), which suggested that mitochondrial structure was damaged in fibrotic mammary tissue. The protein level of TFAM in mammary tissue was significantly lower than that in the control group (Figure 4A,D, *p* < 0.01), which suggested that mitochondrial function was abnormal in fibrotic mammary tissue. In addition, the levels of COI, COII, and COIV mRNA in fibrotic mammary tissue were significantly lower than those in the control group (Figure 4G–I, *p* < 0.01), and this result suggests that mitochondrial energy metabolism is abnormal in fibrotic mammary tissue. In addition, in fibrotic cow mammary gland tissue, the ROS level was significantly higher than that in healthy mammary gland tissue (Figure 4J, *p* < 0.01). The above results indicate that the mitochondrial structure in fibrotic mammary tissue was damaged and its function was impaired, which was not conducive to the balance of energy homeostasis and is conducive to the occurrence and development of fibrosis.

### 3.5. Changes of Inflammatory and Fibrotic Phenotypes in TGF-β1-Induced bMECs

**Figure 5:**
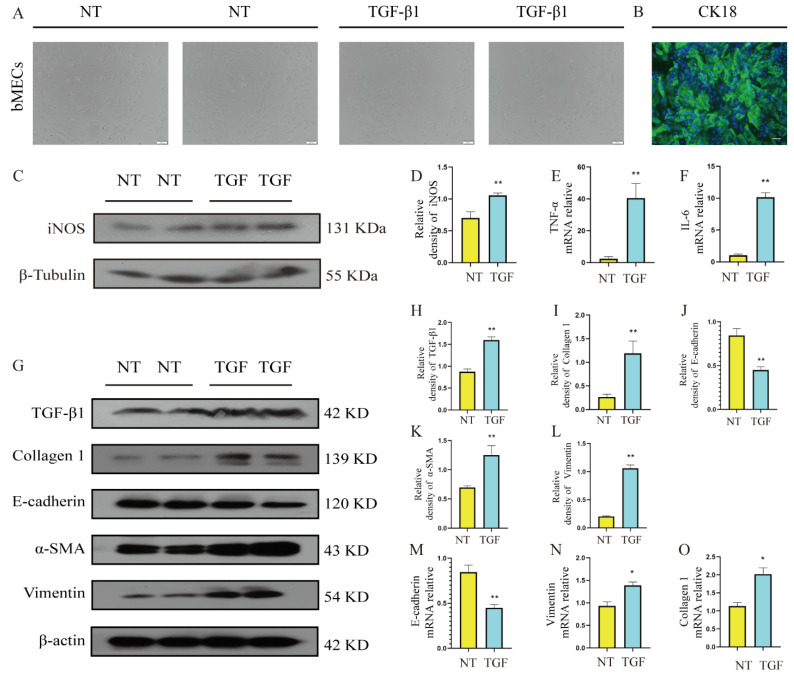
Changes of inflammatory and fibrotic phenotypes in TGF-β1-induced bovine mammary epithelial cells (bMECs). TGF-β1 was added to DMEM culture without FBS to stimulate bMECs for 48 h, and then the cells were collected for subsequent experiments. (**A**) The cell morphology of bMECs stimulated by TGF-β1 for 48 h. (**B**) Immunofluorescence staining of CK18, a specific marker of bMECs, scale bar 50 μM. (**C**) The protein levels of iNOS and β-actin in bMECs. (**D**) Quantification of iNOS protein levels in bMECs, relative to β-actin. (**E**,**F**) The mRNA expression levels of TNF-α, IL-6 in bMECs. (**G**) The protein levels of collagen 1, E-cadherin, α-SMA, vimentin and β-actin in bMECs. (**H**–**L**) The corresponding quantitative analysis of protein bands in (**G**). (**M**–**O**) The mRNA expression levels of E-cadherin, collagen 1, vimentin in bMECs. Data were analyzed by the unpaired *t*-test method. The data error is presented in the form of mean ± SEM. The results of the experiment are the result of three independent repeated experiments. * *p* < 0.05, ** *p* < 0.01 compared with the control group.

In the previous in vivo results, it has been confirmed that TGF-β1 was abundantly secreted in mastitis tissue, which may be a potential cause of fibrotic lesions in mastitis tissue. In order to further explore and clarify the pathogenesis, this experiment was based on the bMECs induced by TGF-β1 in vitro to determine whether the EMT process of bMECs drives the occurrence and development of fibrosis by promoting the fibrotic phenotype. First, the immunofluorescence results showed that bMECs abundantly expressed the epithelial cell-specific marker keratin 18 (CK-18), which proved the stability and scientificity of the bMECs used in this study (Figure 5B). In addition, after TGF-β1 stimulation of bMECs for 48 h, the cell morphology changed, the characteristics of epithelial cells disappeared, and the oval paving cobblestone-like shape changed to a long spindle shape, which marked the occurrence of epithelial–mesenchymal transition (Figure 5A). Further studies showed that the gene and protein levels of vimentin and collagen 1, and the protein levels of TGF-β1 and α-SMA, were significantly increased (Figure 5G–O, *p* < 0.01). In addition, in the process of TGF-β1 stimulation of bMECs, iNOS protein levels were significantly increased (Figure 5C,D, *p* < 0.01) and IL-6 and TNF-α mRNA levels of inflammatory cytokines were significantly increased (Figure 5E,F, *p* < 0.01). The above findings suggest that TGF-β1 stimulates bMECs to produce higher levels of inflammation, and promotes a significant increase in the fibrotic phenotype, which in turn drives the progression of mammary fibrosis.

### 3.6. Changes of Mitochondrial Damage in TGF-β1-Induced bMECs

**Figure 6:**
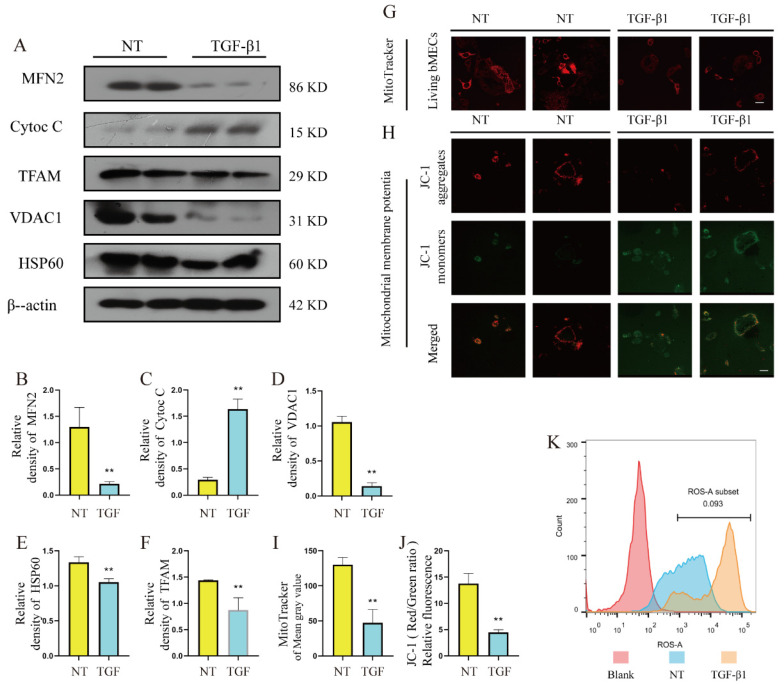
Changes of mitochondrial damage in TGF-β1-induced bMECs. TGF-β1 was added to DMEM culture without FBS to stimulate bMECs for 48 h, and then the cells were collected for subsequent experiments. (**A**) The protein levels of MFN2, Cyto C, TFAM, VDAC1, HSP60, and β-actin in bMECs. (**B**–**F**) Quantification of MFN2, Cyto C, TFAM, VDAC1, HSP60 protein levels in bMECs. (**G**) MitoTracker Red CMXRos staining of bMECs, the faint red light means that the number and function of mitochondria are reduced, scale bar 60 μM. (**H**) Detection of mitochondrial membrane potential of bMECs, stained with JC-1s, scale bar 40 μM. (**I**) Average fluorescence intensity analysis of MitoTracker in (**G**). (**J**) Quantitative analysis of mitochondrial membrane potential detection with JC-1. (**K**) Flow cytometry detects the production of ROS produced by cells. Data were analyzed by the unpaired *t*-test method. The data error is presented in the form of mean ± SEM. The results of the experiment are the result of three independent repeated experiments. ** *p* < 0.01 compared with the control group.

In order to explore the mitochondrial damage of bMECs stimulated by TGF-β1, the mitochondrial-related damage phenotype was detected in this experiment, and the following experimental results were obtained. The results showed that TGF-β1 significantly reduced the protein levels of HSP60 and VDAC1, which suggested that we reduced the number of mitochondria (Figure 6A,D,E, *p* < 0.01). TGF-β1 significantly reduced the protein level of MFN2, suggesting that mitochondrial fusion is impaired and mitochondrial fragmentation occurs (Figure 6A,B, *p* < 0.01). Compared with the control group, TGF-β1 significantly induced a significant increase in the protein level of Cytoc C (Figure 6A,C, *p* < 0.01), indicating that mitochondrial contents were released and mitochondria were damaged. In addition, it was found that TGF-β1 significantly induced a decrease in mitochondrial membrane potential (Figure 6G,H), a large amount of ROS accumulation (Figure 6K), and the protein level of TFAM was significantly reduced, indicating mitochondrial dysfunction (Figure 6A,F, *p* < 0.01). The above results further prove that bMECs were involved in the occurrence and development of mammary fibrosis after being stimulated by TGF-β1, the mitochondrial structure was damaged, and the function was impaired.

### 3.7. Effects of Inhibition of ROS Accumulation on TGF-β1-Induced Fibrotic Phenotype of bMECs

**Figure 7:**
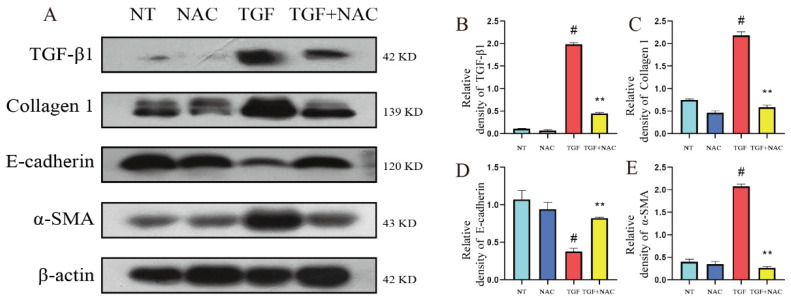
Effects of inhibition of ROS accumulation on TGF-β1-induced fibrotic phenotype of bMECs. The ROS scavenging reagent NAC (3 mM) was added to DMEM, then 1 h later TGF-β1 was added to DMEM to stimulate bMECs for 48 h. (**A**) The protein levels of TGF-β1, collagen 1, E-cadherin, α-SMA, vimentin. (**B**–**E**) Quantification of TGF-β1, collagen 1, E-cadherin, α-SMA, vimentin protein levels in bMECs. Data were analyzed by one-way ANOVA method. The data error is presented in the form of mean ± SEM. The results of the experiment are the result of three independent repeated experiments. # *p* < 0.01 compared with the control group, ** *p* < 0.01 compared with the TGF-β1 group.

In order to explore the effect of scavenging ROS on the fibrotic phenotype of bMECs induced by TGF-β1, this experiment detected the changes of the mitochondrial phenotype of bMECs after NAC scavenged ROS, and obtained the following experimental results. The results showed that compared with the TGF-β1 group, the TGF-β1 + NAC treatment significantly alleviated the TGF-β1-induced increase in TGF-β1, collagen 1, and α-SMA protein levels in bMECs (Figure 7A,C,E) and significantly alleviated the TGF-β1-induced reduction of E-cadherin protein levels in bMECs (Figure 7A,D). The experimental results in Figure 7 indicate that inhibiting ROS accumulation can effectively alleviate the TGF-β1-induced increase in the fibrotic phenotype of bMECs, which may be a potential therapeutic approach to alleviate mammary fibrosis.

## 4. Discussion

Mammary gland fibrosis in dairy cows has caused huge economic losses to animal production. Therefore, it is necessary to explore the pathogenesis of mammary gland fibrosis and find effective treatments. In response to the above problems, this study explored the relationship between mastitis, mammary fibrosis, and mitochondrial damage at two levels of in vivo and in vitro experiments.

Inflammation is often an important initiating factor that induces fibrosis [25], and the process of fibrosis is often accompanied by strong inflammatory stimulation [25]. This study found that there was a higher level of inflammation in the cows’ mammary gland tissue in the model group, so there may be a certain degree of mammary gland fibrosis in the mammary gland tissue. Therefore, we detected the related phenotypic proteins of fibrosis in mastitis tissue. α-SMA and vimentin are markers of fibrosis activation. The large expression of these two proteins in epithelial cells often means that the migration ability of epithelial cells and the ability of cells to secrete ECM are enhanced [1]. E-cadherin is a calcium-dependent transmembrane protein involved in cell–cell adhesion and has important physiological functions such as maintaining epithelial cell polarity and maintaining the mammary gland barrier [26]. Decreased levels of E-cadherin protein often imply the disappearance of epithelial cell characteristics and initiation of the EMT process, which exacerbates the process of mammary fibrosis [1]. Activated fibroblasts and EMT secrete a large amount of ECM, which is also the main reason for the process of fibrosis. Collagen is the main component of the extracellular matrix. The significant increase in the level of collagen protein marks the occurrence and development of the fibrosis process [1,4]. TGF-β1 is a transforming growth factor involved in the fibrosis process by regulating cell proliferation and migration, differentiation, ECM production, and immune regulation. The massive secretion of TGF-β1 often leads to the continuous occurrence and development of fibrosis, which was regarded as the main inducer of fibrosis [27,28]. Therefore, exogenous recombinant protein TGF-β1 is often used as an inducer for in vitro fibrosis models [27,29]. The Masson staining results in this study showed that there were also a large number of collagen fibers deposited in the tissue, and collagen fibers were the main component of the ECM, which indicated that the mammary tissue had severe mammary fibrosis [1]. In addition, the gene and protein results also showed that the above fibrotic phenotypes were significantly increased in mastitis tissue, and similarly, the above fibrotic phenotypes were also significantly increased in the TGF-β1-stimulated bMEC model, further confirming the presence of strong mastitis tissue. There was a positive correlation between mastitis and mammary fibrosis.

Mitochondria are the site of cellular aerobic respiration and also the energy factory of cells. The structural integrity and normal function of mitochondria are important factors for maintaining cellular homeostasis [9]. However, mitochondria are relatively fragile organelles. When they bear great metabolic pressure, they are easily damaged by stimulating factors [30]. Studies have shown that bMECs have mitochondrial damage and dysfunction in the process of mastitis [11,12], and mitochondrial damage is often positively correlated with fibrosis [13,31]. Therefore, the present study examined the phenotypic proteins and mitochondrial membrane potential changes associated with fibrosis in mastitis tissue and TGF-β1-stimulated bMECs. Decreased levels of the mitochondrial fusion protein MFN2 imply a weakened mitochondrial fusion capacity, and studies have shown that the loss of MFN2 leads to aggravation of pulmonary fibrosis [32]. Cyto C is an embedded protein located on the outside of the inner mitochondrial membrane and is an important protein in the process of the respiratory chain. The increase in the level of Cyto C protein means that the mitochondria are damaged. Studies have shown that the release of Cyto C is related to the process of apoptosis-related fibrosis [33]. Mitochondrial transcription factor A (TFAM) plays an important role in maintaining mitochondrial DNA stability [34]. Studies have shown that a significant decrease in the protein level of TFAM represents a malfunction of mitochondrial function [35], and promoting the increase in TFAM protein level is conducive to alleviating the dysfunction caused by mitochondrial damage. VDAC1 and HSP60 have relatively highly conserved DNA sequences, so they are often used as mitochondrial markers. In this experiment, they were used to reflect changes in the number of mitochondria [36]. The results of this experiment showed that the above mitochondrial indexes changed significantly in mastitis tissue, mitochondrial damage was obvious, and mitochondrial function was impaired. The above experimental results were also supported in TGF-β1-induced bMECs, the mitochondrial membrane potential was significantly reduced, and there was a large amount of ROS accumulation. ROS is a typical endogenous stimulator after mitochondrial injury, and it may be the main stimulator of the development of mammary gland fibrosis.

ROS produced by mitochondria is a strong oxidizing substance, which is often closely related to the occurrence and development of diseases as a typical stimulus. Studies have shown that higher levels of ROS lead to the initiation of disease phenotypes [37]. For example, high levels of ROS are closely related to the process of fibrosis [16] and, in the process of hepatic fibrosis, ROS promotes the activation of hepatic stellate cells, induces massive proliferation of hepatic stellate cells, increases their ability to secrete ECM, and finally leads to the occurrence of hepatic fibrosis [38,39]. Therefore, timely removal of a large amount of accumulated ROS often has a mitigating effect on the fibrosis process [16,17]. The results of this experiment confirmed that scavenging the accumulation of ROS significantly reversed the increase in the fibrotic phenotype of bMECs induced by TGF-β1. Therefore, the results of this experiment suggest that inhibiting the accumulation of ROS may be an effective treatment for alleviating mammary fibrosis.

**Figure 8:**
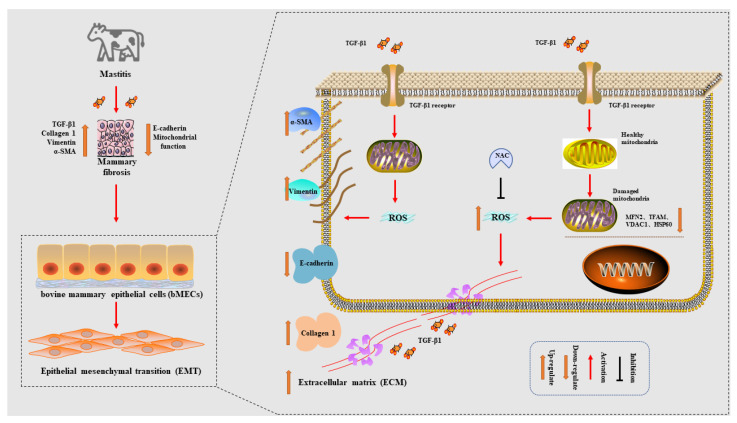
Schematic diagram of the experimental mechanism. The fibrotic phenotype of mammary gland tissue was significantly elevated in cows with mastitis, and mastitis disease was positively correlated with mammary fibrotic lesions. In an in vitro and in vivo model of cow mammary fibrosis, bMECs have impaired mitochondrial structure and dysfunction. Inhibiting the accumulation of ROS effectively alleviates the elevated fibrotic phenotype, which may be a potential therapeutic approach to alleviate mammary fibrosis.

In conclusion, the fibrotic phenotype of mammary gland tissue of dairy cows with mastitis was significantly increased, and mastitis disease was positively correlated with mammary fibrotic lesions. In an in vitro and in vivo model of cow mammary fibrosis, bMECs have impaired mitochondrial structure and dysfunction. Inhibiting the accumulation of ROS effectively alleviates the elevated fibrotic phenotype, which may be a potential therapeutic approach to alleviate mammary fibrosis, but specific and in-depth studies are needed to further confirm and support this notion (Figure 8).

## Figures and Tables

**Table 1:** Nutrient composition of the diets.

Item	Measurement
Ingredient (%)	
Corn silage	40.00
Corn	35.00
Wheat bran	8.00
Soybean meal	5.00
Sunflower	8.00
NaCl	1.00
Premix	1.80
NaHCO_3_	1.20
Total	100.00
Nutrient composition (% of DM)	
NE L (MJ/kg)	6.70
CP	15.20
NDF	33.45
ADF	17.20
NFC	40.40
Ca	0.70
P	0.50

Provided the following per kilogram of diet: vitamin A 200,000 IU, vitamin D 70,000 IU, vitamin E 1000 IU, Fe 2000 mg, Cu 600 mg, Zn 2400 mg, Mn 1300 mg, I 6 mg, Co 7 mg.

**Table 2:** Classification of clinical mastitis.

Mastitis Grade (1–3)	Grading Standards	
	Milk Block Content in Milk	Symptom
Grade 1 mastitis	Milk contains a small number of lumps	No obvious symptoms such as redness, swelling, heat, and pain in the mammary area
Grade 2 mastitis	Milk contains more lumps	Obvious separation of water and milk, slight redness, swelling, heat, and pain in the mammary area
Grade 3 mastitis	Milk contains a lot of lumps	The cows showed symptoms of general discomfort, increased body temperature, watery milk, and severe redness, swelling, heat, and pain in the milk area

**Table 3:** Healthy cows.

ID	Type of Infection	Mastitis Grade	Somatic Cell Count	Parity of Calves
160298	No natural and human-made infection	No mastitis	2–200,000/mL	2
160557	No natural and human-made infection	No mastitis	2–200,000/mL	3
160072	No natural and human-made infection	No mastitis	2–200,000/mL	2
160125	No natural and human-made infection	No mastitis	2–200,000/mL	3
160097	No natural and human-made infection	No mastitis	2–200,000/mL	2
160557	No natural and human-made infection	No mastitis	2–200,000/mL	2
172507	No natural and human-made infection	No mastitis	2–200,000/mL	3
175871	No natural and human-made infection	No mastitis	2–200,000/mL	3
170210	No natural and human-made infection	No mastitis	2–200,000/mL	2
170956	No natural and human-made infection	No mastitis	2–200,000/mL	2

**Table 4:** Mastitis cows.

ID	Type of Infection	Mastitis Grade (1–3)	Somatic Cell Count	Parity of Calves
160033	Natural infection mainly caused by Escherichia coli	Grade 2 mastitis	>200,000/mL	3
160491	Natural infection mainly caused by Escherichia coli	Grade 2 mastitis	>200,000/mL	3
166047	Natural infection mainly caused by Escherichia coli	Grade 2 mastitis	>200,000/mL	2
166077	Natural infection mainly caused by Escherichia coli	Grade 2 mastitis	>200,000/mL	3
170363	Natural infection mainly caused by Escherichia coli	Grade 2 mastitis	>200,000/mL	2
170440	Natural infection mainly caused by Escherichia coli	Grade 2 mastitis	>200,000/mL	2
170447	Natural infection mainly caused by Escherichia coli	Grade 2 mastitis	>200,000/mL	3
180310	Natural infection mainly caused by Escherichia coli	Grade 2 mastitis	>200,000/mL	2
180526	Natural infection mainly caused by Escherichia coli	Grade 2 mastitis	>200,000/mL	3
180843	Natural infection mainly caused by Escherichia coli	Grade 2 mastitis	>200,000/mL	3

**Table 5:** The primer sequences.

Item	Primer (5′-3′)	Product Length (bp)
*a-SMA (Forward)*	GAAGCCCAGCCGAGAACTTT	194
*a-SMA (Reverse)*	TCCCACCATCACTCCCTGAT	
*Collagen 1 (Forward)*	ACTGAAACCCCCGAAAAGCC	220
*Collagen 1 (Reverse)*	GTGGGTCTTCAAGCAAGTGG	
*COI (Forward)*	TATGGACTGGAACGGGAGAG	162
*COI (Reverse)*	GCTTCTTTGGACACTTGAGCA	
*COII (Forward)*	CAGAACCTGATGCTTTGTGC	106
*COII (Reverse)*	ACTCGTCAACCCTCTCCTTG	
*COIV (Forward)*	ATCTCGGGTTTTTGGGTTGC	334
*COIV (Reverse)*	GGGTGGTGGTCCAGGTTCTC	
*E-cadherin (Forward)*	AAAGAGAGTGGAAGTGCCCG	255
*E-cadherin (Reverse)*	GCAGGTGGAGAACCATTGTC	
*IL-1β (Forward)*	ATGAAGAGCTGCATCCAACACCTG	107
*IL-1β (Reverse)*	ACCGACACCTGCCTGAAG	
*IL-6 (Forward)*	GCCTTCACTCCATTCGCTGTCTC	117
*IL-6 (Reverse)*	AAGTAGTCTGCCTGGGGTGGTG	
*TNF-α (Forward)*	CTGGCGGAGGAGGTGCTCTC	85
*TNF-α (Reverse)*	GGAGGAAGGAGAAGAGGCTGAGG	
*Vimentin (Forward)*	GTCCAAGTTTGCTGACCTCTC	134
*Vimentin (Reverse)*	TAGTCCCTTTGAGCGCATCC	
*β-actin (Forward)*	GCCCTGAGGCTCTCTTCCA	101
*β-actin (Reverse)*	GCGGATGTCGACGTCACA	

## Data Availability

Data is available only on request due to privacy restrictions. The data are not publicly available due to the privacy of the participants of the study.
